# Reproductive health service use and social determinants among the floating population: a quantitative comparative study in Guangzhou City

**DOI:** 10.1186/s12913-014-0502-8

**Published:** 2014-10-30

**Authors:** Huan Liu, Qi Wang, Zuxun Lu, Junan Liu

**Affiliations:** Department of Social Medicine and Health Management, School of Public Health, Tongji Medical College, Huazhong University of Science and Technology, No.13, Hangkong Road, Wuhan, Hubei Province PR China; Department of Epidemiology and Biostatistics, School of Public Health, Tongji Medical College, Huazhong University of Science and Technology, No.13, Hangkong Road, Wuhan, Hubei Province PR China; Xiangyang Central Hospital, No.13, Jingzhou Street, Xiangyang, Hubei Province PR China

**Keywords:** Floating population, Residential population, Reproductive health, Family planning services, Socioeconomic status

## Abstract

**Background:**

The World Health Assembly has pledged to achieve universal reproductive health (RH) coverage by 2015. Therefore, China has been vigorously promoting the equalisation of basic public health services (i.e. RH services). The floating population (FP) is the largest special group of internal migrants in China and constitutes the current national focus. However, gaps exist in the access of this group to RH services in China.

**Methods:**

A total of 453 members of the FP and 794 members of the residential population (RP) aged 18 to 50 years from five urban districts in Guangzhou City were recruited to participate in a cross-sectional survey in 2009. Information on demographics and socioeconomic status (SES) were collected from these two groups to evaluate the utilisation of RH knowledge and skills and family planning services (FPS), and to identify social determinants.

**Results:**

The proportion of individuals with low SES in the FP (19.2%) was higher than that in the RP (6.3%) (*P* <0.001). Of the FP, 9.7% to 35.8% had no knowledge of at least one skill, a proportion higher than the counterpart values (6.2% to 27.5%) for the RP (*P* <0.05). The frequency of FPS use among the FP and RP was low. However, FPS use was higher among the FP than among the RP (3.51 vs. 2.99) (*P* =0.050). Logistic regression analysis was used to analyse the social determinants that influence FPS use in the FP and RP. The factors that affect FPS utilisation of the RP included SES (OR =4.652, 95% CI =1.751, 12.362), whereas those of the FP excluded SES.

**Conclusions:**

The FPS use of the FP in Guangzhou City was higher under equalised public health services. However, a need still exists to help the FP with low SES to improve their RH knowledge and skills through access to public RH services.

## Background

Reproductive health (RH) was initially proposed by the World Health Organization Special Programme of Research in Human Reproduction (WHO/HRP) in 1988. The International Conference on Population and Development held in Cairo in 1994 officially defined RH as a state of complete physical, mental and social well-being, not merely the absence of disease, in all matters relating to the reproductive system and to its function and processes. The definition of RH was the core content of the “*Platform for Action”.* The conference likewise became a milestone in RH history [1]. The World Health Assembly held in 1995 re-emphasised the importance of a global RH strategy and advanced the international health objective of achieving universal access to RH by 2015 [2]. Three of the eight Millennium Development Goals adopted by the United Nations in 2000 were directly related to reproductive and sexual health (i.e. improving maternal health, reducing child mortality, and fighting HIV/AIDS, malaria, and other diseases) [3]. In 2004, WHO announced the global RH strategy once again [4] along with the recommendations for monitoring RH at the national level [5].

RH is not only related to human reproduction and development, but also to several kinds of major diseases and social problems. Therefore, RH is vital to the sustainable development of population, society, and economy, and is an important as well as indispensable part of the overall health of humans. It not only affects the current society, but also directly influences the future of human society. Hence, enjoying RH is a common right of everyone.

Floating population (FP) is a terminology used to describe a group of people who reside in a given population for a certain amount of time and for various reasons, but are not generally considered as part of the official census count. Based on the ‘Report on the development of floating population in China’ [6], the total FP size in China was nearly 230 million in 2013, a number that constitutes 17% of the total national population. Approximately 23 million members of the FP have been living in Guangdong Province for over half a year. Guangdong is the top FP residence, with a total FP population of over 28 million, accounting for roughly 12% of the entire FP in China. Four million members of the FP in Guangdong reside in Guangzhou City [7]. In China, households are divided into urban and rural households based on the geographical location of the official residence of the householder and on the occupation of his/her parents. Different household types enjoy different types of social welfare. As a transmigration group between urban and rural areas, the FP has a fairly low educational level and poor living and working environments. Their condition leads to poor health and presents an urgent need for public health services. In particular, the FP is vulnerable to public health problems, especially to those related to RH. As receivers of RH services, the FP is a disadvantaged group. A study in Guangzhou City reported that the rate of antenatal examination for the residential population (RP) was 99.72% in 2003, the rate of prenatal care was 86.4%, and the rate of hospital delivery was 98.4%. In comparison, the rate of antenatal examination for the FP was only 74.53%, the rate of prenatal care was only 43.1%, and the hospital delivery rate was only 75.3% [8]. Another survey in Zhejiang Province showed that the rate of postpartum visit for the FP was 85.84% and the system administration rate was 45.64%, whereas the rate of postpartum visit for the RP was 96.34% and the system administration rate was 96.34%. Such findings reveal that the FP falls behind the RP in terms of prenatal care, hospital delivery, postpartum visit, and system administration. Some studies have reported that since the FP only moved into their current location, the provision of family planning services (FPS) is not ideal, and the rate of access to FPS is low, reaching only 25% to 30% [9].

As an important part of the Millennium Development Goals, equal access to RH services has been recognised and accepted worldwide. In recent years, China has vigorously promoted the equalisation of basic public services in family planning, which enables the FP to enjoy the same propaganda, service, and management of FPS as the RP. Therefore, the present survey took the RP as the control and performed a comparative analysis between the control and the FP. The objective was to identify gaps and deficiencies, as well as to provide evidence for decision making to improve RH public services for the FP. In October 2010, China vigorously carried out pilot work to promote the equalisation of a basic public health service (i.e. FPS), which aims to cover all of the FP in pilot cities [10]. In the current study, data on demographic and socioeconomic status (SES) characteristics were collected from both FP and RP to evaluate RH knowledge and skills and the utilisation of RH services, as well as to identify their social determinants. The findings are expected to provide evidence to assist the improvement of RH public services for the FP.

## Methods

This study was approved by the Ethics Committee of Tongji Medical College, Huazhong University of Science and Technology (IRB No: FWA00007304). Before the investigation, a written informed consent form regarding the method, purpose, and meaning of this survey was provided to the respondents. If the respondents wished to take part in the survey, they were asked to affix their signatures on the page. Written informed consent forms were obtained from all respondents prior to the investigation.

### Subjects of the study

The criteria for eligible FP participants were as follows: (1) a rural-to-urban migrant aged 18 to 50 years, (2) residing at Guangzhou City for more than six months, and (3) can provide oral informed consent. The excluding criterion was the inability to read or answer the study questionnaires (e.g. dementia, difficulties with the language). The criteria for eligible RP participants were as follows: (1) member of a registered household in Guangzhou City, and aged 18 to 50 years, (2) residing at Guangzhou City for more than six months, and (3) can provide oral informed consent.

### Face-to-face interview

By reviewing existing literature and consulting relevant experts, we develop a self-made questionnaire that contained four components: (1) personal demographic characteristics; (2) household demographic characteristics; (3) self-reported knowledge and RH skill level, RH needs, and RH information approach; and (4) utilisation of RH services in different health care settings. The questionnaire was filled by the respondents themselves. In the case of an illiterate subject, a trained investigator filled the questionnaire with the answers of the respondent. Completing the questionnaire required approximately 20 minutes. All the completed questionnaires were collected immediately by the investigators. If the respondents can use basic RH knowledge and skills, we judge their skills level as good. If they have limited RH knowledge and skills, we judge their skills as midlevel. If the respondents have no RH knowledge and skills, we judge their skill level as poor.

### Sampling strategy

Five administrative zones, namely, Baiyun, Huangpu, Yuexiu, Liwan, and Haizhu, were selected out of the 12 administrative zones or counties in Guangzhou. These zones represent new or old regions as well as developing or developed economies. All communities in the five zones were classified into two categories based on their economic situation. One community was randomly selected from each category. Finally, a total of 10 communities were selected, from which the subjects were randomly recruited. A total of 1,300 questionnaires were distributed and 1,247 valid questionnaires were returned, generating a response rate of 95.92%. The sample used in this survey constituted 453 members of the FP and 794 members of the RP.

### SES classification standard

Relevant research data from the WHO confirmed that health status, health behaviour, health service accessibility, and health service utilisation were related with SES (i.e. education, income, occupation), and vary based on race, ethnicity, and gender [6,11]. Sociologists commonly use SES as a means to predict individual behaviour. Therefore, in the present study, we used this index to identify the relationship of SES with FPS utilisation.

In this study, demographic characteristics, SES, and other measures of FP and RP were analysed. The SES is designated to the position or rank of a person or group in society. The SES is a comprehensive assessment of individual educational level, income, occupation, wealth, living area, and so on [12-14]. Education, income, and occupation are the most commonly used SES indicators [15]. In the present study, we selected personal monthly income, occupation, and educational level as SES indicators. The variables of these indicators were categorised and assigned to corresponding scores following the list in Table 1. The total of the assignment scores for the three variables ranged from 3 to 15. To facilitate further data analysis, we assumed a total score of 3 to 6 to indicate low SES, 7 to 11 for middle-level SES, and 12 to 15 for high SES.

**Table 1:** **Educational level, income, and occupation assignments**

***Personal monthly income (RMB)***	***Educational level***	***Occupation***	**Score**
≥4000	University or above	Administrative institutions or enterprises	5
3000 ~ 3999	Senior middle school or polytechnic school	Businessmen	4
2000 ~ 2999	Junior middle school	Service industries	3
1000 ~ 1999	Elementary school	Retirees with retired wages	2
<1000	Other else	Awaiting job assignment/laid-off workers	1

### Statistical methods

To test the statistical significance between groups, t-test was used for measurement variables and chi-square test was used for categorical variables. Binary logistic regression analysis with selection method was likewise used to identify the dependence of FPS use on independent variables. These variables included age, sex, marital status, number of women and children in the family, SES, social security, RH needs, location of the Community Health Center (CHC) or Family Planning Service Center (FPSC), RH care awareness, participation in RH lectures, and RH knowledge and skills.

Logistic regression analyses were performed to examine the correlating factors of FPS utilisation. Differences exist between the FP and RP in terms of SES, and the family planning work of the government for the FP and RP varies as well. Hence, we performed logistic regression separately for FP and RP to obtain the different influencing factors. We used enter methods to select variables that are correlated with FPS utilisation. Outcome variables included the FPS use and odds ratios (ORs) and their 95% confidence intervals (CIs). The inclusion *P* value was 0.05, and the removal *P* value was 0.10. EpiData version 3.0 was used to construct the database. Statistical software SPSS version 17.0 was utilised to perform the statistical analyses.

## Results

### Demographic and SES characteristics

Table 2 shows the demographic and SES characteristics of the respondents. A total of 1,247 respondents aged 18 to 50 years were examined. The mean (SD) respondent age was 35.3 (8.01) years. The FP had a mean age (SD) of 33.0 (7.85) years, which was lower than the counterpart value of 36.6 (7.82) years for the RP respondents (*P* <0.001). Up to 65.1% of the FP were aged between 18 and 36 years old. The proportion of unmarried subjects among the FP (18.5%) was larger than that among the RP (11.7%) (*P* =0.001). The proportion of the FP that engaged in social health insurance was 60.9%, which was smaller than that of the RP (83.8%) (*P* <0.001). Furthermore, 21.9% of the FP and 30.6% of the RP participated in commercial health insurance (*P* =0.001). No difference was observed in the gender composition between the FP and RP, as well as in the composition of households that enjoy the minimum living guarantee. With regard to the SES, 83.2% of the FP were at the middle SES level or below, and 19.2% of the FP were at the low SES level. Meanwhile, 93.7% of the RP were at the middle SES level or above, while only 6.3% of the RP were at the low SES level. The average annual household income of the RP was RMB 45,600, which was higher than that of the FP (RMB 34,000).

**Table 2:** **Demographic characteristics and SES of** FP **and RP**

**Demographics**	***FP(%) N = 453***	***RP(%) N = 794***	***P*** **-value**
**Age (years)**			<0.001*
18–24	73 (16.1)	55 (06.9)	
25–36	222 (49.0)	345 (43.5)	
37–50	158 (34.9)	394 (49.6)	
**Gender**			0.501
Male	111 (24.5)	209 (26.3)	
Female	342 (75.5)	585 (73.7)	
**Marital status**			0.001*
Married	84 (18.5)	93 (11.7)	
Unmarried	369 (81.5)	701 (88.3)	
**Minimum living guarantee enjoyment**			0.168
No	408 (90.1)	734 (92.4)	
Yes	45 (09.9)	60 (07.6)	
**Social insurance**			<0.001*
Not involved	177 (39.1)	129 (16.2)	
Involved	276 (60.9)	665 (83.8)	
**Commercial insurance**			0.001*
Not involved	354 (78.1)	551 (69.4)	
Involved	99 (21.9)	243 (30.6)	
**SES**			<0.001*
Low	87 (19.2)	50 (06.3)	
Middle	290 (64.0)	522 (65.7)	
High	76 (16.8)	222 (28.0)	
**Average annual household income (mean ± std) (×RMB 10000)**	3.40 ± 4.01	4.56 ± 4.58	0.011*

*P ≤0.05 indicates a statistical significance.

### RH knowledge and skills

Table 3 presents the findings on RH knowledge and skills among the FP and RP. Compared with the RP, a smaller percentage of the FP possessed RH knowledge about newlywed health (47.9% vs. 59.1%), prevention and control of sexually transmitted diseases (STDs) (36.4% vs. 43.1%), health care of pregnant women and puerpera (41.3% vs. 54.4%). The proportion of the FP that had no knowledge about such types of information was larger than that of the RP. However, no difference was observed between the FP and RP in terms of the frequency of participation in RH lectures. Up to 61.8% of the FP and 56.2% of the RP reported having never participated in RH lectures.

**Table 3:** **Level of RH knowledge and skills among FP and RP**

**RH knowledge and skills**	***FP N = 453(%)***	***RP N = 794(%)***	***P*** **-value**
**RH skills for Pregnancy test**			0.002*
Good	158 (34.9)	349 (44.0)	
Middle	203 (44.8)	328 (41.3)	
Poor	92 (20.3)	117 (14.7)	
**Contraception**			<0.001*
Good	231 (51.0)	486 (61.2)	
Middle	167 (36.9)	274 (34.5)	
Poor	55 (12.1)	34 (04.3)	
**Cleaning reproductive tracts**			0.007*
Good	181 (40.0)	381 (48.0)	
Middle	228 (50.3)	363 (45.7)	
Poor	44 (09.7)	50 (06.3)	
**Maternal nutrition during pregnancy**			0.001*
Good	92 (20.3)	205 (25.8)	
Middle	241 (53.2)	445 (56.0)	
Poor	120 (26.5)	144 (18.1)	
**Miscarriage prevention**			<0.001*
Good	82 (18.1)	173 (21.8)	
Middle	209 (46.1)	403 (50.8)	
Poor	162 (35.8)	218 (27.5)	
**Prenatal education**			<0.001*
Good	86 (19.0)	180 (22.7)	
Middle	213 (47.0)	428 (53.9)	
Poor	154 (34.0)	186 (23.4)	
**Safe sex**			<0.001*
Good	186 (41.1)	417 (52.5)	
Middle	217 (47.9)	328 (41.3)	
Poor	50 (11.0)	49 (06.2)	
**RH knowledge about health care of pregnant women**			<0.001*
Good	187 (41.3)	432 (54.4)	
Middle	212 (46.1)	300 (37.8)	
Poor	54 (11.9)	62 (07.8)	
**Prevention and control of STD infection**			0.016*
Good	165 (36.4)	342 (43.1)	
Middle	209 (46.1)	353 (44.5)	
Poor	79 (17.4)	99 (12.5)	
**Newlywed health**			<0.001*
Good	217 (47.9)	469 (59.1)	
Middle	181 (40.0)	276 (34.8)	
Poor	55 (12.1)	49 (06.2)	
**Attendance in RH lectures**			0.056
Yes	173 (38.2)	348 (43.8)	
No	280 (61.8)	446 (56.2)	

*P ≤0.05 indicates a statistical significance.

Similarly, in comparison with the RP, a smaller percentage of the FP was equipped with RH skills for pregnancy test (34.9% vs. 44.0%), contraception (51.0% vs. 61.2%), cleaning genital tracts (40.0% vs. 48.0%), maternal nutrition during pregnancy (20.3% vs. 25.8%), miscarriage prevention (18.1% vs. 21.8%), early education for foetus (19.0% vs. 22.7%), and safe sex (41.1% vs. 52.5%). The proportion of the FP that reported no RH skills was larger than that of the RP.

### FPS utilisation

Table 4 shows the utilisation of FPS based on service providers in the latest year for both the FP and RP. The table indicates that the average number of times the FP received FPS was higher than that of the RP. However, no significant difference existed between the FP and RP in terms of the presence of hospitals, CHC, MCHB, and FPSC. The average number of times that the FP and RP received FPS from the FPSC, 1.87 and 1.73, respectively, was relatively higher than that for the other locations. The average number of times that the FP used FPS in the CHC was 0.67, which was higher than that of the RP (0.48) (*P* =0.006).

**Table 4:** **Mean (mean ± std) of times that subjects utilise FPS, grouped by service providers**

**Service providers**	***FP***	***RP***	***P*** **-value**
Hospitals	0.57 ± 1.145	0.46 ± 0.986	0.079
MCHB	0.39 ± 0.929	0.32 ± 0.845	0.206
CHC	0.67 ± 1.299	0.48 ± 0.930	0.006*
FPSC	1.87 ± 2.915	1.73 ± 2.530	0.364
Total	3.51 ± 4.758	2.99 ± 4.044	0.050*

*P ≤0.05 indicates a statistical significance.

### Logistic regression analysis of FPS utilisation

The results of logistic regression analysis on the relationship of FPS use with the demographics and SES of the FP and RP are presented in Table 5. The factors that influenced the FPS use of the FP included gender (*OR* =0.208), marital status (*OR* =0.267), RH needs (*OR* =3.246), participation in RH lectures (*OR* =3.113), pregnancy test skills (*OR* =4.981), health awareness (*OR* =5.303), and knowledge about the location of FPS institutions (*OR* =2.141). Meanwhile, the factors that influenced the FPS use of the RP included SES (*OR* =4.652), contraceptive skills (*OR* =2.570), gender (*OR* =0.456), marital status (*OR* =0.299), RH needs (*OR* =1.663), participation in RH lectures (*OR* =1.987), and number of children under five years in the family (*OR* =0.557).

**Table 5:** **Social determinants of FPS use of FP and RP**

**Social determinants of FPS use**	**FP**		**RP**	
	***OR(*** **95% ** ***CI)***	***P*** **-value**	***OR(*** **95% ** ***CI)***	***P*** **-value**
**Gender**		<0.001*		<0.001*
Female	1		1	
Male	0.208(0.108 ~ 0.401)		0.208(0.108 ~ 0.401)	
**Marital status**		0.001*		0.001*
Married	1		1	
Unmarried	0.267(0.120 ~ 0.596)		0.267(0.120 ~ 0.596)	
**SES level**		0.459		0.459
High	1		1	
Middle	1.600(0.744 ~ 3.441)		1.600(0.744 ~ 3.441)	
Low	1.692(0.673 ~ 4.255)		1.692(0.673 ~ 4.255)	
**RH needs**		<0.001*		<0.001*
No	1		1	
Yes	3.246(1.683 ~ 6.260)		3.246(1.683 ~ 6.260)	
**RH skill for contraception**		0.655		0.655
Poor	1		1	
Middle	1.240(0.359 ~ 4.280)		1.240(0.359 ~ 4.280)	
Good	1.582(0.445 ~ 5.617)		1.582(0.445 ~ 5.617)	
**Attendance in RH lectures**		<0.001*		<0.001*
No	1		1	
Yes	3.113(1.920 ~ 5.048)		3.113(1.920 ~ 5.048)	
**RH care awareness**		0.015*		0.015*
Poor	1		1	
Middle	7.183(1.790 ~ 28.821)		7.183(1.790 ~ 28.821)	
Good	5.303(1.227 ~ 22.918)		5.303(1.227 ~ 22.918)	
**RH skill for pregnancy test**		<0.001*		<0.001*
Poor	1		1	
Middle	2.837(1.396 ~ 5.767)		2.837(1.396 ~ 5.767)	
Good	4.981(2.395 ~ 10.359)		4.981(2.395 ~ 10.359)	
**Knowledge of the FPSC location**		0.049*		0.049*
No	1		1	
Yes	2.141(1.002 ~ 4.572)		2.141(1.002 ~ 4.572)	
**Number of children under five years in the family**	0.949(0.523 ~ 1.722)	0.863	0.949(0.523 ~ 1.722)	0.863

*P ≤0.05 indicates a statistical significance.

## Discussion

Our findings show that the FP is younger, has a lower SES level and insurance rate, and reports more RH needs than the RP. Nearly half of the FP in our study were sexually active young adults aged 25 to 36 years, living far away from their hometowns, and had sexual partners or no fixed sexual partners. Such subjects were also characterised by sexual liberality, high incidence of premarital sex, and poor awareness of safe sex. In addition, their low SES level, poor self-protective consciousness, and lack of knowledge on RH and contraception increased their risks of premarital pregnancy, abortion, STDs, and HIV infection. According to Zheng et al. [16], the incidence of premarital pregnancy and extramarital pregnancy among the FP is increasing, and the rate of induced abortion for unwanted pregnancies is high. In a survey, Zhang [17] reported that 25% of unmarried members of the FP exhibit reckless sexual behaviours. Such behaviours often increase the prevalence of accidental pregnancies and induced abortion among female members of the FP. These unsafe sex behaviours have also been significant risk factors for the spread of STDs and AIDS. Sun et al. [18] reported that the RH care-seeking behaviours of the FP are mainly instigated by the need to seek health care services and medical treatment for reproductive disorders. In particular, the FP encountered serious issues about unplanned pregnancies and health care of pregnant women and puerpera, as well as the prevalence of reproductive illnesses as a result of their behaviours. A considerable number of FP members engaged in a so-called 3D (i.e. dirty, dangerous, and difficult) career, which is characterised by the lack of essential economic and medical security. Several members of the FP included in our study were awaiting job assignments or were unemployed and had no income. Therefore, they were vulnerable to poverty when they arrived in the city. The ignorance of such members of the FP on RH issues also aggravated the problem of reproductive safety, which merits urgent attention.

The FP generally had some RH knowledge and skills. However, their level of such RH knowledge and skills was lower than that of the RP. Zhou [19] revealed that the FP was equipped with less knowledge on contraception than the household population in Wuhan City. On the one hand, this situation may be a consequence of the imperfect management system, based on which the FP members did not compulsively receive FPS provided by the CHC, MCHB, or other public health institutions. Therefore, we hypothesise that the FP is inadequately served and cared for (i.e. insufficient health education for the FP) in many aspects of RH care. The FP members mainly served as a registered population in a city, despite the existence of a relevant policy that suggests that the FP should enjoy the same services as those as the RP, with birth control as the principal goal [20]. On the other hand, the high risks of RH issues among the FP are associated with the low educational level, frequent migrations, impeded information channels, and poor RH skills and knowledge of the population. Based on the KAP model of health promotion theory, knowledge is the foundation of attitude and behavioural change [21]. People who have health knowledge and who adopt positive and correct beliefs as well as attitudes tend to conduct healthy behaviours and foster healthy habits. Women with inadequate RH knowledge may therefore be unaware of the risks of gynaecological diseases, and may refrain from seeking medical treatment because of the belief that such diseases will not affect their lives.

The Chinese government has implemented a family planning program to control the excessive population growth and improve population quality and health. The program is regarded as a basic national policy of China. China has attached great importance to FPS for a long time and has established the specialised FPSC to promote the FPS use of the FP. Moreover, the CHCs are fundamental providers of the basic public health and medical services included in the primary health care system. The FP is familiar with CHCs and is more inclined to seek medical help or treatment from such centres in case of RH disorders. According to Chen Hui, 73.3% of the FP hope to receive the FPS provided by FPS centres, whereas 23.1% hope to receive such services provided by CHCs [22]. Sun et al. [23] found that low-level medical institutions are given priority by the FP in seeking RH-related medical services. Other reports also show that [24] women constituted a majority in seeking community-based RH service. Therefore, the Chinese government should strengthen the community service system and improve the accessibility of public RH services based on the characteristics of the FP.

In this study, the factors that influence the FPS use of residents included gender, marital status, RH knowledge and skills, and SES. This result is consistent with those of a Canadian study [25] that reported educational level, gender, poverty, and migration as the major social factors related to RH. Another local study [17] indicated that the main influencing factors of RH service use among the FP were educational level, marital status, and RH knowledge. In the current study, people with RH needs naturally received FPS more than those without such needs. Compared with females, males reported less FP use, a finding also confirmed by Debra et al. [26]. Furthermore, RH care awareness considerably influenced the use of RH services for both the FP and RP. According to Peng, the RH status is closely associated with self-care awareness among rural women in China [27]. This phenomenon can be explained by the fact that people with a strong sense of self-care tend to seek medical treatment as soon as they are ill, and they focus closely on their own health, especially in terms of reproductive and contraception health. Sufficient RH awareness likewise contributes to the generation of correct RH behaviours [28].

The SES is found to be closely related to the use of RH services. El-Kak indicated that women from low-income families are more likely to use subsidised RH services [29]. Poor people typically experience high levels of unmet family planning needs, whereas such unmet needs gradually decrease among the rich as a result of their increased FPS use. Women with an unmet need are those who are fecund and sexually active but do not use any contraception method and report a lack of preference for more children or a desire to delay the birth of their next child [30]. In Malawi, the rate of FPS use was approximately 20% and 36% among married women at the bottom and top SES levels respectively. On the contrary, based on a report in 2007, the FPS use was relatively equal across all SES groups in the Philippines and in Bangladesh. Evidence demonstrates decreasing inequalities in the FPS use among various SES groups [24].

In China, governments are implementing the equalisation policy of public RH services to promote equity in RH use among the FP. Government-purchased FPS are provided freely to individuals in need regardless of SES level, thereby equalising FPS use among the FP. As for the RP, the low SES group faces more RH problems and needs, therefore resulting in higher FPS use. The low SES group in the RP belongs to the vulnerable group as the FP in society. Hence, the government should also pay more attention to this group to improve RH, and consequently, to improve social management and safeguard social stability. Chen et al. reported that the Chinese Yi people of high SES have a better RH status compared with those of low SES [9]. Another piece of evidence indicates that women of low SES are more likely to suffer reproductive tract infections than other women [31]. Therefore, the low SES population is a group that requires significant FPS in China.

In China, sex is generally considered to be an embarrassing topic for the unmarried population. Only married women tend to talk about experiences and information about sex and contraception and have free access to contraceptive measures of the local family planning departments [28]. Few people discuss relevant issues on contraception and sex with unmarried women. Therefore, the unmarried population has limited knowledge about RH, not to mention the use of RH services. Our findings also indicated that the larger the number of children aged less than five years is in a family, the less frequent the FPS use. This finding is consistent with the study of Tangcharoensathien, in which women with two or more children are reported to receive less essential obstetric services compared with those who have less than two children [23]. This phenomenon is attributed to the need of parents to spend considerable time and energy in caring for their children, therefore reducing their opportunities to use FPS.

## Conclusions

The FP in Guangzhou City is characterised by a lower SES level and insurance rate, more RH needs, and a lower level of self-protective consciousness compared with the RP. Meanwhile, the FPS use of the FP is better than that of the RP because of government attention. The FP requires the provision of more RH care services (i.e. health education). These findings provide evidence that can assist decision makers in bridging these coverage gaps. Achieving a truly universal RH coverage would certainly improve the RH consciousness of the FP in China.

## Abbreviations

FP: Floating population
RP: Residential population
RH: Reproductive health
FPS: Family planning service
SES: Socioeconomic status
CHC: Community Health Center
FPSC: Family Planning Service Center
MCHB: Maternal and Child Health Bureau
STDs: Sexually transmitted diseases

## References

[1] UNFPA (1995). Report of the International Conference on Population and Development, Cairo, 5–13 September 1994. Programme of Action.

[2] WHO (2010). To accelerate progress towards the attainment of international development goals and targets. Progress Report 2010.

[3] UN (2000). The Millennium Development Goals.

[4] WHO (2004). To accelerate progress towards the attainment of international development goals and targets. Progress Report 2004.

[5] WHO (2008). National-level monitoring of the achievement of universal access to reproductive health: conceptual and practical considerations and related indicators. Report of a WHO/UNFPA Technical Consultation.

[6] WHO (2000). The World Health Report 2000: Health System: Improving Performance.

[7] Guangdong Survey Research Center (2011). Perfecting the urban FP’s management system to build a harmonious Guangdong. The Investigation Report of Guangdong Affairs.

[8] Chen Q, Zeng FL, Wang P (2004). The investigation and analysis of maternal health status and influencing factors for 777 women of the floating population in Guangzhou City. J Guangzhou Med.

[9] Wu JQ, Li YY, Ye JF, Zhang YF, Zhao R, Zheng XY, Zhan SK, Chen JP, Yang TZ (2011). The effects of contraceptive intervention on the demand for family planning services in the floating population. Int J Reprod Health/Fam Plann.

[10] National Population and Family Planning Commission: **49 pilot cities started the work of FP’s equalization of public services**. 2013. [http://www.nhfpc.gov.cn/ldrks/dcyxk/201312/555b03ccd12549f39ce2f253061e4799.shtml]

[11] Adler NE, Chesney MA, Boyce WT, Susan F, Leonard SS (1993). Socioeconomic inequalities in health No easy solution. JAMA.

[12] Gulliford MC, Sedgwick JE, Pearce AJ (2003). Cigarette smoking, health status, socio-economic status and access to health care in diabetes mellitus: a cross-sectional survey. BMC Health Serv Res.

[13] Liu XN, Gao WL, Yan H (2014). Measuring and decomposing the inequality of maternal health services utilization in Western Rural China. BMC Health Serv Res.

[14] Xiao YY, Zhao NQ, Wang H, Zhang J (2013). Association between socioeconomic status and obesity in a Chinese adult population. BMC Public Health.

[15] Liu LH, Tang JX (2004). The effect of Socio-economic status on residents’ health equity. Chin Health Econ.

[16] Zheng LX, Zhu JM, Tian PL, Chen Y, Song YJ, Chen YS (2000). The risk factors of reproductive health among floating unmarried young women workers in Guangzhou City. South Popul.

[17] Zhang M (2008). The Research of Factors that Influence the Floating Population’s Reproductive Health Service Utilization.

[18] Sun RX, Sun YK, Xu YL (2008). The related care-seeking behaviors and influencing factors of reproductive health among floating population. Matern Child Health Care Chin.

[19] Zhou HF, Hou QJ, Lu ZX (2012). The comparison analysis of contraceptive knowledge and behavior between census register population and floating population in Wuhan City. Chin J Soc Med.

[20] Tang J (2012). The Explore Research of Floating Unmarried Women’s Reproductive Health Status, Influencing Factors and Community-Based Interventions.

[21] Grunseit A, Kippax S (1997). Impact of HIV and sexual health education on the behavior of young people. A Review Update.

[22] Chen H (2011). Discussion on the wishes and influencing factors of family planning services for the rural floating population. Chin Health Ind.

[23] Tangcharoensathien V, Tantivess S, Teerawattananon Y, Auamkul N, Jongudoumsuk P (2002). Universal coverage and its impact on reproductive health services in Thailand. Reprod Health Matters.

[24] USAID (2007). Inequalities in the use of family planning and reproductive health Services. Health Policy Initiative.

[25] Shaw D (2009). Access to sexual and reproductive health for young people: bridging the disconnect between rights and reality. Int J Gynaecol Obstet.

[26] Debra K, Bruce A, Molly F, Gabrielle H, Jessica G (2008). Evaluation of a community-based sexual health intervention for young adult Latino and African-American men. J Mens Health.

[27] Peng C (2012). The Research of Rural Women’s Reproductive Health Status and Its Influencing Factors.

[28] Mei MF (2005). Floating Population’s Reproductive Health Promotion.

[29] El-Kak F, Khawaja M, Salem M, Zurayk H (2009). Care-seeking behavior of women with reproductive health problems from low-income areas of Beirut. Int J Gynaecol Obstet.

[30] WHO (2010). Sexual and reproductive health: Unmet need for family planning. Global Health Observatory.

[31] Zeng G (2002). Comprehensive demonstration project of controlling reproductive tract infections. South China J Prev Med.

